# Incomplete Vogt-Koyanagi-Harada Syndrome Presenting With Sunset Glow Fundus, Vitiligo, Preserved Vision, and Incidental CA 19-9 Elevation

**DOI:** 10.7759/cureus.98983

**Published:** 2025-12-11

**Authors:** Nicolas Nicolaou, Antigoni Koukkoulli, Loukiana Tsierkezou

**Affiliations:** 1 General/Vascular Surgery, Addenbrooke's Hospital, Cambridge University Hospitals NHS Foundation Trust, Cambridge, GBR; 2 Neuro-Ophthalmology and Oculoplastics, Pantheo Eye Center, Limassol, CYP; 3 Medical Retina and Uveitis, Pantheo Eye Center, Limassol, CYP

**Keywords:** complete vogt-koyanagi-harada (vkh), elevated ca 19-9, granulomatous panuveitis, sunset glow fundus, vitiligo

## Abstract

Vogt-Koyanagi-Harada (VKH) disease is a T cell-mediated autoimmune attack on melanocytes in the eyes, ears, skin, and central nervous system. It is characterized by granulomatous panuveitis and often results in significant visual loss. We report a 60-year-old woman referred for suspected chorioretinal inflammation following one month of treatment for anterior uveitis. Remarkably, visual acuity was preserved at 6/6 in both eyes. There was no history of ocular surgery or trauma. Examination revealed trace anterior chamber cells and mild anisocoria. Choroidal folds were present without serous retinal detachment, and the inflammation resolved with topical corticosteroids. At five months, progressive choroidal hypopigmentation resulted in a sunset glow fundus. As no neurological or auditory features were present, the diagnosis initially suggested probable VKH. However, the later development of a single forehead vitiligo patch led to the diagnosis of incomplete VKH. During the acute uveitic stage, a benign liver mass was identified alongside elevated liver enzymes and CA 19-9 tumor marker, which subsequently normalized. Ophthalmologists should be aware of atypical presentations of VKH, particularly in older adults, where systemic symptoms may be mild, absent, or delayed. Vision can remain preserved in the absence of serous retinal detachment. Although the precise triggers of VKH remain unclear, immunological activation has been associated with factors such as immune checkpoint inhibitors, vaccinations, viral infections, and coexisting autoimmune diseases. In this case, a coincidental liver mass was observed, with a transient elevation in tumor marker and liver enzymes. Further case reports are needed to clarify whether such immunoinflammatory changes play a role in initiating autoimmune VKH or represent incidental findings.

## Introduction

Vogt-Koyanagi-Harada (VKH) is a rare autoimmune disorder characterized by bilateral granulomatous panuveitis, which may cause significant visual loss [1-4]. Cytotoxic T cells are thought to target melanocyte-rich tissues in the ocular, auditory, vestibular, and central nervous systems [1,2]. Precise triggers remain unclear, although autoimmune, pharmacological, and immunological associations have been reported [3-5]. VKH predominantly affects individuals from pigmented ethnic backgrounds, with higher prevalence in middle-aged women, and is strongly associated with HLA-DRB1 and HLA-DR4 alleles [1-5]. The condition progresses through prodromal, acute uveitic, convalescent, and recurrent stages [3-6]. Bilateral ocular involvement without prior trauma or surgery, with neurological/auditory and integumentary signs, such as vitiligo, alopecia, or poliosis, defines complete VKH. Incomplete VKH is diagnosed when only one systemic feature is present. Probable VKH refers to isolated ocular findings without systemic association [3-5].

VKH in older adults is less common and may present atypically with limited systemic manifestations. Although significant visual loss can occur in VKH and requires urgent treatment, we report a case of incomplete VKH in an older adult in whom visual acuity remained preserved. Systemic signs were minimal, limited to a single late-onset vitiligo patch and a sunset glow fundus (progressive choroidal depigmentation), which contributed to delayed diagnosis. The cause of VKH was unknown; however, during the acute uveitic phase, liver function tests (LFTs) were transiently elevated. Tumor marker CA 19-9 was also raised, later returning to normal, and a benign liver mass not previously documented was identified. The clinical significance of these hepatic findings remains uncertain.

This case emphasizes the importance of recognizing VKH in older patients even when visual function is preserved and extraocular features are subtle or delayed. Further reports are also needed to clarify whether VKH may be associated or triggered by immunoinflammatory activity or whether this was a coincidental finding.

## Case presentation

A 60-year-old Mediterranean female presented with persistent bilateral eye redness and blurry vision. She had been treated for anterior uveitis for one month but was subsequently referred with suspected chorioretinal inflammation. She recalled an episode of malaise, mild chest discomfort, and an insect bite upon returning from abroad. No ocular trauma, infection, surgery, or systemic signs, such as headache, tinnitus, hearing loss, or meningismus, were reported.

On examination, visual acuity was 6/6 in both eyes. Pupillary reflexes were normal, with mild anisocoria and no relative afferent pupillary defect (RAPD). Early lens opacities were also observed. Slit-lamp examination revealed trace anterior chamber cells (+/-OD >OS). Intraocular pressures were normal (OD: 14 mmHg, OS: 15 mmHg). Bilateral choroidal folds were observed on fundal examination, with no evidence of vasculitis or vitritis.

**Figure 1:**
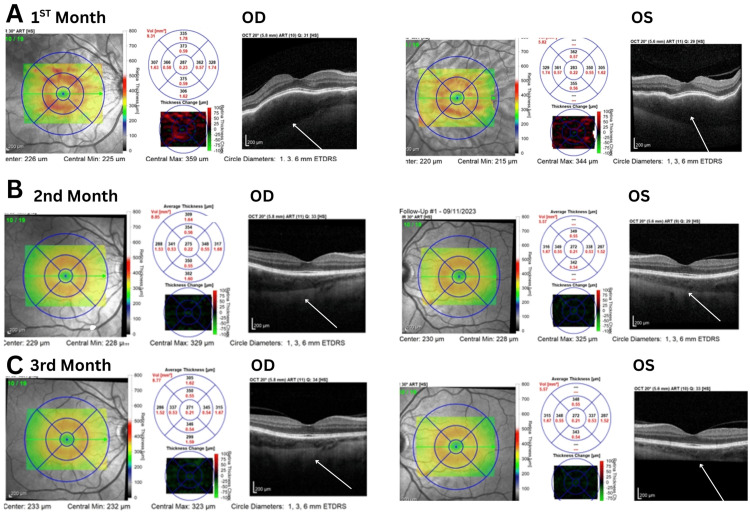
Enhanced depth imaging optical coherence tomography (EDI-OCT). Enhanced depth imaging optical coherence tomography (EDI-OCT) at one, two, and three months demonstrated progressive resolution of choroidal thickening (highlighted by white arrows) and associated inflammatory changes in both eyes following topical corticosteroid therapy. No exudative retinal detachment was observed, and visual acuity remained preserved throughout the follow-up period. ETDRS: Early Treatment Diabetic Retinopathy Study; ART: automated real-time; OCT: optical coherence tomography

**Figure 2:**
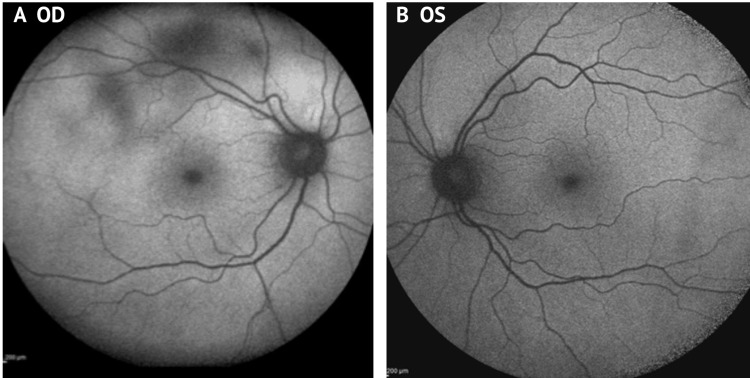
Fundus autofluorescence (FAF) of the right eye (OD) and left eye (OS). The right eye (OD) shows areas of decreased autofluorescence (dark patches) corresponding to underlying choroidal thickening.

Enhanced depth imaging optical coherence tomography (EDI-OCT) demonstrated diffuse choroidal thickening without evidence of serous retinal detachment. Fundus autofluorescence (FAF) revealed diffuse hyperautofluorescence in the right eye (OD). Indocyanine green angiography (ICG) was not performed due to iodine allergy (Figures 1A-1C, 2A, 2B).

Medical history included cholecystectomy and liver hemangioma, with no autoimmune disease. Prior to VKH onset, targeted surveillance due to a strong family history of cancer (glioblastoma, lung, abdominal) included laboratory tests and whole-body imaging, all of which were unremarkable.

Laboratory screening for inflammatory, infectious, autoimmune, and granulomatous diseases was unremarkable. Serologies for Borrelia and Rickettsia (prompted by an insect bite), antinuclear antibody (ANA), antineutrophil cytoplasmic antibody (ANCA), rheumatoid factor (RF), systemic lupus erythematosus (SLE) panel, Mantoux test for tuberculosis, sarcoidosis work-up, and venereal disease research laboratory (VDRL) test for syphilis were negative. Complete blood count (FBC), angiotensin-converting enzyme (ACE), erythrocyte sedimentation rate (ESR), and thyroid function tests (TFTs) were normal. However, CA 19-9 was elevated to 81.18 IU/mL, with mildly elevated liver enzymes, whereas CA125 and CEA were normal.

Orbital MRI showed bilateral optic nerve tortuosity without abnormal enhancement. Brain, vestibulocochlear MRI, and chest X-ray were unremarkable. However, non-contrast CT of the chest, abdomen, and pelvis revealed a 3 cm low-attenuation lesion in the right hepatic lobe, with features suggestive of a benign process. Further characterization was limited as IV contrast was not given due to an allergy. Electroretinography (ERG) revealed normal full-field and multifocal responses, excluding photoreceptor or neural retinal dysfunction seen in paraneoplastic retinopathy, though photopic ERGs were borderline.

Initial management with topical dexamethasone eye drops four times daily bilaterally, together with a short course of cyclopentolate 1% three times daily OD, led to a marked reduction in anterior uveitis and choroidal inflammation within one month. By three months, choroidal thickness had decreased significantly, and dexamethasone was tapered to twice daily.

**Figure 3:**
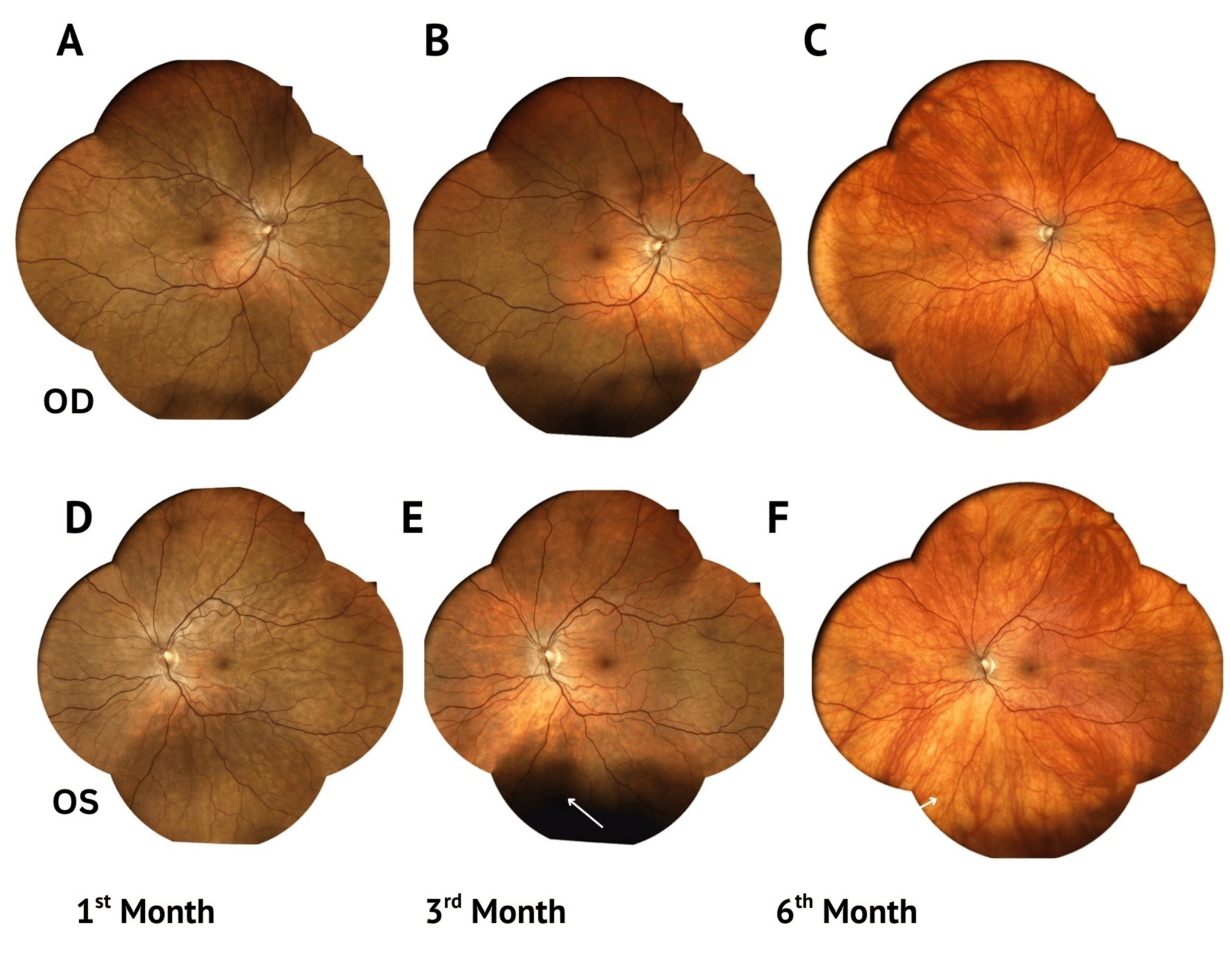
Progression to sunset glow fundus in both eyes. Serial true-color fundus photographs showing progressive choroidal depigmentation in the right (OD) (A-C) and left (OS) (D-F) eyes over six months, demonstrating the development of a characteristic sunset glow fundus.

Five to six months later, a sunset glow fundus developed, though visual acuity remained 6/6 bilaterally (Figures 3A-3F). Additionally, a small forehead patch consistent with vitiligo appeared. CA 19-9 and liver function tests (LFTs) spontaneously returned to normal levels (43.26 IU/mL). No reports of elevated tumor markers in VKH were found in the literature.

## Discussion

Vogt-Koyanagi-Harada (VKH) syndrome is a rare cause of bilateral panuveitis. Key differentials include acute posterior multifocal placoid pigment epitheliopathy (APMPPE), birdshot chorioretinopathy, multifocal choroiditis with panuveitis (MCP), syphilis, sarcoidosis, tuberculosis, and intraocular lymphoma [4,6].

Our patient, an elderly female who initially presented with ocular symptoms, was thought to have probable VKH but was later reclassified as having incomplete VKH following the appearance of a single vitiligo patch five months later. Systemic signs are less frequent in older adults [4]. In a large cohort of 1,012 patients, 9% were classified as probable, 71% as incomplete, and 12% as complete VKH [5]. In contrast, Kaza et al. reported 72% probable, 26% incomplete, and only 1% complete VKH in a cohort of 69 elderly patients [3]. This highlights that most elderly cases show only ocular involvement, confirming that systemic features decrease with age [3]. Vitiligo typically occurs in about 40% of patients and usually appears two to three months into the convalescent stage, though it may present later, as observed in this case [1,3,4]. Table 1 summarizes the classification criteria for VKH.

**Table 1:** Diagnostic criteria for Vogt-Koyanagi-Harada (VKH) disease. Complete VKH disease requires criteria A, B, and C, at least one early-phase feature from criterion D, at least one late-phase feature from criterion E, and both the following systemic manifestations: (A) neurological/auditory and (B) integumentary. Incomplete VKH disease presents similarly to complete VKH but includes only one systemic manifestation (either neurological/auditory or integumentary, but not both). Probable VKH disease presents as complete VKH without any systemic manifestations. Complete VKH accounts for 12%, incomplete for 71%, and probable for 9% of cases [5]. In older adults, probable VKH has been reported in up to 72% of cases, reflecting the lower frequency of systemic features in this age group [3]. This table was created by the authors of this study using information from references [2,5,7,8]. FFA: fluorescein fundus angiography; EDI: enhanced depth imaging; OCT: optical coherence tomography

Ocular manifestations
A. No history of penetrating ocular trauma or intraocular surgery before the onset of uveitis.
B. Bilateral ocular involvement.
C. No evidence of infectious uveitis, systemic rheumatic disease, or other ocular conditions that would account for the presentation.
D. Acute uveitis (early-phase) VKH features.
1. Diffuse choroiditis with exudative retinal detachment.
2. Without serous retinal detachment detected.
3. Choroidal thickening or effusion.
4. Early punctate hyperfluorescence with late subretinal dye pooling on fluorescein angiography (FFA).
5. Hyperfluorescence of the optic disc on FFA.
E. Convalescent (late-phase) VKH features.
1. Moth-eaten hyperfluorescence on FFA.
2. Sunset glow fundus or retinal pigment epithelium (RPE) clumping/migration.
3. Bilateral recurrent granulomatous anterior uveitis.
4. Dalen-Fuchs nodules or multifocal chorioretinal atrophy.
Systemic manifestations
A. Neurological or auditory findings (may occur in the prodromal phase and resolve before presentation).
Meningismus (malaise, fever, headache, nausea, abdominal or neck/back pain or stiffness; headache alone is insufficient), tinnitus, or cerebrospinal fluid pleocytosis.
B. Integumentary changes occurring after ocular or neurological involvement.
Alopecia, poliosis or vitiligo.

Recognition of VKH in older adults may be delayed, as it typically affects individuals aged 20-50 years, with only 10% of cases occurring after this age group [1,4,5]. Common age-related findings, such as tinnitus, hearing loss, and cataract, may further contribute to the delay in diagnosis [4]. The prodromal phase often mimics a viral illness, with headache, fever, dizziness, periorbital pain, and meningismus. Auditory symptoms include tinnitus and hearing loss [1-3]. Our patient experienced only mild malaise and chest discomfort, highlighting variability in presentation. The acute uveitic stage typically develops within two weeks and is characterized by bilateral granulomatous panuveitis [1-5]. In this case, only mild anterior uveitis presented.

Mild anisocoria was observed, although pupillary reflexes remained normal. This may be associated with involvement of the ciliary ganglion or short ciliary nerves, where inflammation can reduce baseline parasympathetic tone by affecting the postganglionic fibers innervating the intraocular muscles [6].

Vision is often significantly impaired in VKH due to exudative retinal detachment, which occurs in over 78% of cases [1,3]. Multimodal imaging in our case demonstrated bilateral choroidal inflammation without serous retinal detachment, preserving visual acuity at 6/6 bilaterally, an uncommon finding [2].

The convalescent phase typically begins several weeks after the acute stage, with resolution of chorioretinal inflammation following corticosteroid therapy (Figures 1A-1C) [1,2]. Our case demonstrates the formation of a sunset glow fundus at one, three, and six months, capturing intermediate changes that are seldom illustrated in other reports (Figures 3A-3F). Sunset glow fundus, reported in 44-57.7% of cases, has been associated with poorer visual prognosis and retinal dysfunction even after treatment [1,3,4]. Electroretinography (ERG) responses frequently show reduced photopic and scotopic amplitudes [4]. In our patient, however, vision remained preserved even after the development of sunset glow fundus, with both full-field and multifocal ERG responses within normal limits. Photopic ERGs, however, were at the lower limit of normal, suggesting possible early cone involvement without functional impairment.

The precise triggers of VKH remain unclear. Reported associations include vaccinations (hepatitis B, COVID-19), pharmacological agents, such as interferon-alpha and ribavirin for hepatitis C, immune checkpoint inhibitors (nivolumab), and BRAF/MEK inhibitors (encorafenib and binimetinib) [7-12]. Increased susceptibility has been linked to HLA-DRB1 and HLA-DR4 alleles, as well as to autoimmune conditions including Graves’ disease, type 1 diabetes, rheumatoid arthritis, and autoimmune thyroid disease [11-14]. These associations share common mechanisms of immune dysregulation that may activate cytotoxic T cells targeting melanocyte-rich tissues of the eyes, ears, and skin, thereby contributing to VKH.

Our case highlights a combination of findings, including abnormal LFTs, elevated CA 19-9, and a hepatic lesion not previously reported. CA 19-9 is a tumor marker commonly measured in the evaluation of pancreatic and gastrointestinal malignancies; however, elevations may also occur in benign conditions, including hepatic, pancreatobiliary, and metabolic diseases [15]. Our patient had undergone regular surveillance due to a family history of malignancy, during which tumor marker levels, including CA 19-9, were within normal limits prior to VKH onset. During the acute stage, CA 19-9 was mildly elevated at 81 IU/mL (with normal CEA and CA 125) but normalized after eight months, a course more consistent with benign or localized hepatic involvement rather than malignancy. It is possible that a hepatic inflammatory process contributed to T cell-mediated immune activation and VKH disease.

## Conclusions

Vogt-Koyanagi-Harada (VKH) in older adults often presents with fewer systemic manifestations, and vision may remain unaffected in the absence of serous retinal detachment. Ophthalmologists should consider VKH in atypical presentations with preserved vision, particularly when systemic features, such as cutaneous signs, appear later. In this case, a reactive hepatic lesion with transient CA 19-9 and liver enzyme elevation was noted during the acute uveitic phase; however, the clinical significance of this association remains uncertain. Further reports are needed to determine whether these immunoinflammatory mechanisms contribute to VKH activation or simply represent coincidental findings.
